# Structural, Electronic, and Magnetic Characteristics of Graphitic Carbon Nitride Nanoribbons and Their Applications in Spintronics

**DOI:** 10.1021/acs.jpcc.2c04691

**Published:** 2022-09-15

**Authors:** M. Reza Rezapour

**Affiliations:** Department of Atomic, Molecular and Nuclear Physics, Faculty of Science, Campus de Fuente Nueva, University of Granada, 18071 Granada, Spain

## Abstract

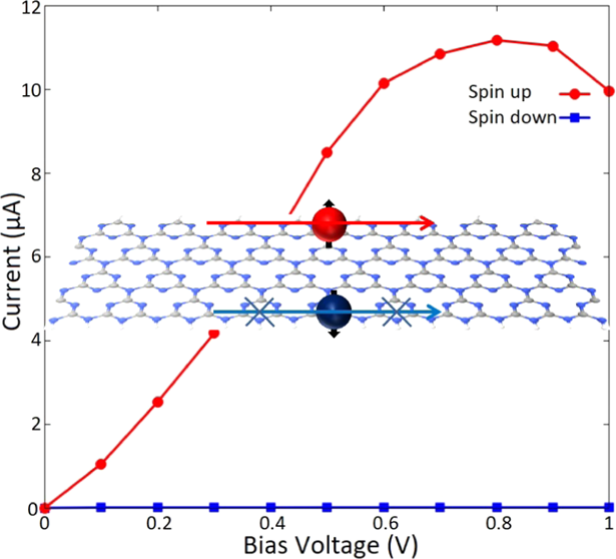

The development of quantum information and quantum computing technology requires special materials to design and manufacture nanosized spintronic devices. Possessing remarkable structural, electronic, and magnetic characteristics, graphitic carbon nitride (g-C_3_N_4_) can be a promising candidate as a building block of futuristic nanoelectronics and spintronic systems. Here, using first-principles calculations, we perform a comprehensive study on the structural stability as well as electronic and magnetic properties of triazine-based g-C_3_N_4_ nanoribbons (gt-CNRs). Our calculations show that gt-CNRs with different edge conformation exhibit distinct electronic and magnetic characteristics, which can be tuned by the edge H-passivation rate. By investigating gt-CNRs with various possible edge configurations and H-termination rates, we show that while the ferromagnetic (FM) ordering of gt-CNRs stays preserved for all of the studied configurations, half metallicity can only be achieved in nanoribbons with specific edge structure under full H-passivation rate. For spintronic application purposes, we also study spin-transport properties of half-metal gt-CNRs. By determining the suitable gt-CNR configuration, we show the possibility of developing a perfect gt-CNR-based spin filter with a spin filter efficiency (SFE) of 100%. Considering the above-mentioned notable electronic and magnetic characteristics as well as its high thermal stability, we show that gt-CNR would be a remarkable material to fabricate multifunctional spintronic devices.

## Introduction

Spintronic devices aim to simultaneously utilize the charge and spin of electrons to deliver, store, and process information.^1,2^ Since the performance efficiency of any spintronic device depends on the spin polarization ratio of the currents provided, finding materials capable of producing 100% spin-polarized current at the Fermi level is a necessity. A half metal, i.e., a material in which only one spin direction possesses metallic characteristics, fully meets this demand.^3,4^ In recent years, tremendous efforts have been devoted to exploring low-dimensional half metals, including organic and inorganic two-dimensional (2D) sheets,^5−8^ as well as one-dimensional (1D) nanowires.^9−12^ In particular, it has been shown that graphene and graphene analogues,^13−15^ which have attracted a great deal of attention in terms of 2D physics and chemistry,^16−22^ may exhibit intriguing spin states on their edges.^23−26^ Graphene nanoribbons (GNRs) having localized electronic edge states^27−30^ also exhibit magnetic features with the finite-size effect.^31^ Owing to the 1D ballistic transport characteristic of GNR, various nanoelectronics and spintronics applications of GNR-based devices have been investigated.^32−39^ Several techniques have been introduced to induce half metallicity in the electronic structure of graphene or GNR such as the application of electric field,^40^ edge modification,^41^ B/N dopants,^42^ and introduction of particular atomic-scale defects.^43^

Recently, g-C_3_N_4_, a 2D semiconductor benefitting from nitrogen doping and structural defect techniques, has attracted considerable scientific interest due to its appealing electronic and magnetic structures, excellent chemical and thermal stability, and environmentally friendly features.^44−46^ The uniform distribution of vacancies in the structure of g-C_3_N_4_ also makes it a highly capable material to trap foreign atoms without forming clusters. This feature makes g-C_3_N_4_ a good candidate for designing nanosize spintronic devices by doping or adsorbing foreign atoms, such as B, C, Al, or transition metals (TM).^47−50^ It also has been shown that g-C_3_N_4_ possesses potential applications in electrocatalysis, photosynthesis, solar energy conversion, bioimaging application, and photocatalytic performance.^51−54^ Various allotropes of g-C_3_N_4_ have been synthesized,^55−60^ among which heptazine g-C_3_N_4_ (gh-C_3_N_4_) and triazine g-C_3_N_4_ (gt-C_3_N_4_) are generally viewed to be the most energetically stable and have been broadly investigated.^54,56,59,61−63^ Although it has been verified that gh-C_3_N_4_ is the most stable modification under ambient conditions,^64^ gt-C_3_N_4_ also has been successfully synthesized starting from dicyanamide.^65^ Despite its porous framework with well-ordered vacancies, g-C_3_N_4_ is a nonmagnetic (NM) material.^47,66^ However, Du et al. showed that the hole injection via replacing a nitrogen atom with a carbon atom can cause the transition of NM to ferromagnetic (FM) phase in the magnetic structure of g-C_3_N_4_.^67^ Several methods have been proposed to induce ferromagnetism in the electronic structure of g-C_3_N_4_ such as introducing defects,^68^ doping external ions,^69^ fluorine dangling bonds,^70^ and boron bonds.^71^ In particular, for spintronics applications, inducing half metallicity is essential. It should also be noted that although plenty of studies have been conducted to investigate the various characteristics of 2D g-C_3_N_4_, despite its experimental realization,^72−74^ there is still no systematic study on characteristics and possible electronic and spintronic applications of 1D g-C_3_N_4_.

Motivated by all of the above-mentioned facts, in the present work, we perform a comprehensive study on the structural, electronic, and magnetic properties of gt-CNRs, the induction of half metallicity in their magnetic structure, and their spin-transport characteristics. To this end, first, we study the possible structural configurations of gt-CNRs in terms of their edge conformations and energetic stability. Next, the possibility of inducing half metallicity in the electronic structures of gt-CNRs is investigated in terms of their edge structure as well as the H-passivation rate. Our calculations indicate that the electronic and magnetic characteristics of gt-CNRs, hence the induction of half metallicity in its electronic structure, depend on both edge hydrogenation and the edge structure of nanoribbons. In this regard, we show that gt-CNRs with a certain edge configuration can undergo a magnetic phase transition, from NM to half metal, by tuning the H-passivation rate. Finally, we study the spin-transport properties of gt-CNRs and show that a half-metal gt-CNR can be employed to develop a perfect spin filter device.

## Computational Methods

Our first-principles calculations are performed based on the density functional theory (DFT). The Vienna ab initio simulation package (VASP)^75^ is employed for geometry relaxations, cohesive energy calculations, and investigation of the electronic and magnetic structures of the systems. The exchange–correlation effects are treated within the form of the generalized gradient approximation (GGA) of Perdew, Burke, and Ernzerhof (PBE).^76^ The electron–ion interactions are described by the plane-augmented wave (PAW) method and the Kohn and Sham orbitals are expanded in a plane wave basis set.^77^ A 500 Ry cutoff energy for the grid-mesh and a *k*-point mesh of 1 × 1 × 64 are employed along the *x*-, *y*-, and *z*-directions. All of the structures are fully relaxed until energies and forces are converged to 10^–5^ eV and 0.01 eV/Å, respectively. DFT combined with nonequilibrium Green’s function (NEGF)^78^ as implemented in the TranSIESTA code^79^ is employed to investigate the spin-transport characteristics of the proposed spin filter device. The spin-dependent transmission is given by
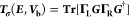
1where **Tr** is the trace, **Γ_L/R_** = ***i***[**∑_L/R_** – **∑_L/R_^†^**] with **∑**_**L/R**_ as the self-energy of the left/right electrode, and ***G*** = [***E*** – ***H*** – **∑_L_** – **∑_R_**]^−1^ is the Green’s function with the scattering region Hamiltonian *H*. Within the Tr[], all quantities implicitly depend on the energy (*E*), *V*_b_, and the spin σ. The current is calculated using the Landauer–Büttiker formalism
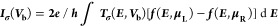
2where ***f***(***E***, **μ**_**L/R**_) is the Fermi–Dirac function with the associated chemical potential **μ**_**L/R**_**=*****E***_**F**_ ± ***V***_**b**_**/2**, which is a shifted value relative to the Fermi level of a neutral system *E*_F_.

## Results and Discussion

**Figure 1:**
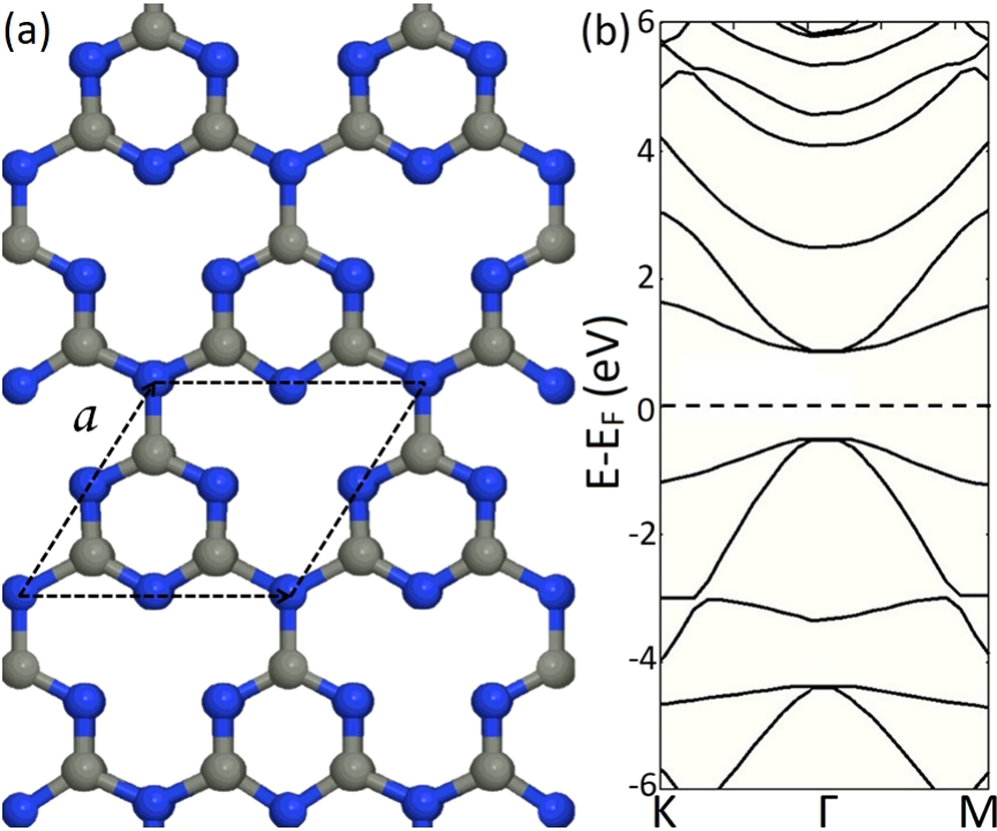
(a) Geometric structures of the gt-C_3_N_4_ monolayer. The lattice vector is indicated by *a* and the unit cell is presented by dashed lines. C and N atoms are depicted in gray and blue. (b) Band structure of the gt-C_3_N_4_ monolayer. The Fermi level is shifted to zero and represented by a dashed line.

Figure 1a illustrates the optimized geometry of a primitive unit cell of a gt-C_3_N_4_ monolayer. The calculated lattice parameter *a* = 4.78 Å is in good agreement with previous studies.^80,81^ The band structure of the introduced unit cell is represented in Figure 1b. It is followed from the calculated band structure that gt-C_3_N_4_ is a direct band gap NM semiconductor with a band gap of *E*_g_ = 1.57 eV. The obtained *E*_g_ is also in agreement with previously reported values.^82,83^ It should be noted that the theoretical *E*_g_, obtained by DFT calculations, heavily depends on the constructed model as well as the employed functional. It has been shown that the calculated *E*_g_ follows the order of HSE06 > GGA-PW91 > GGA-PBE based on the employed functionals.^83^

**Figure 2:**
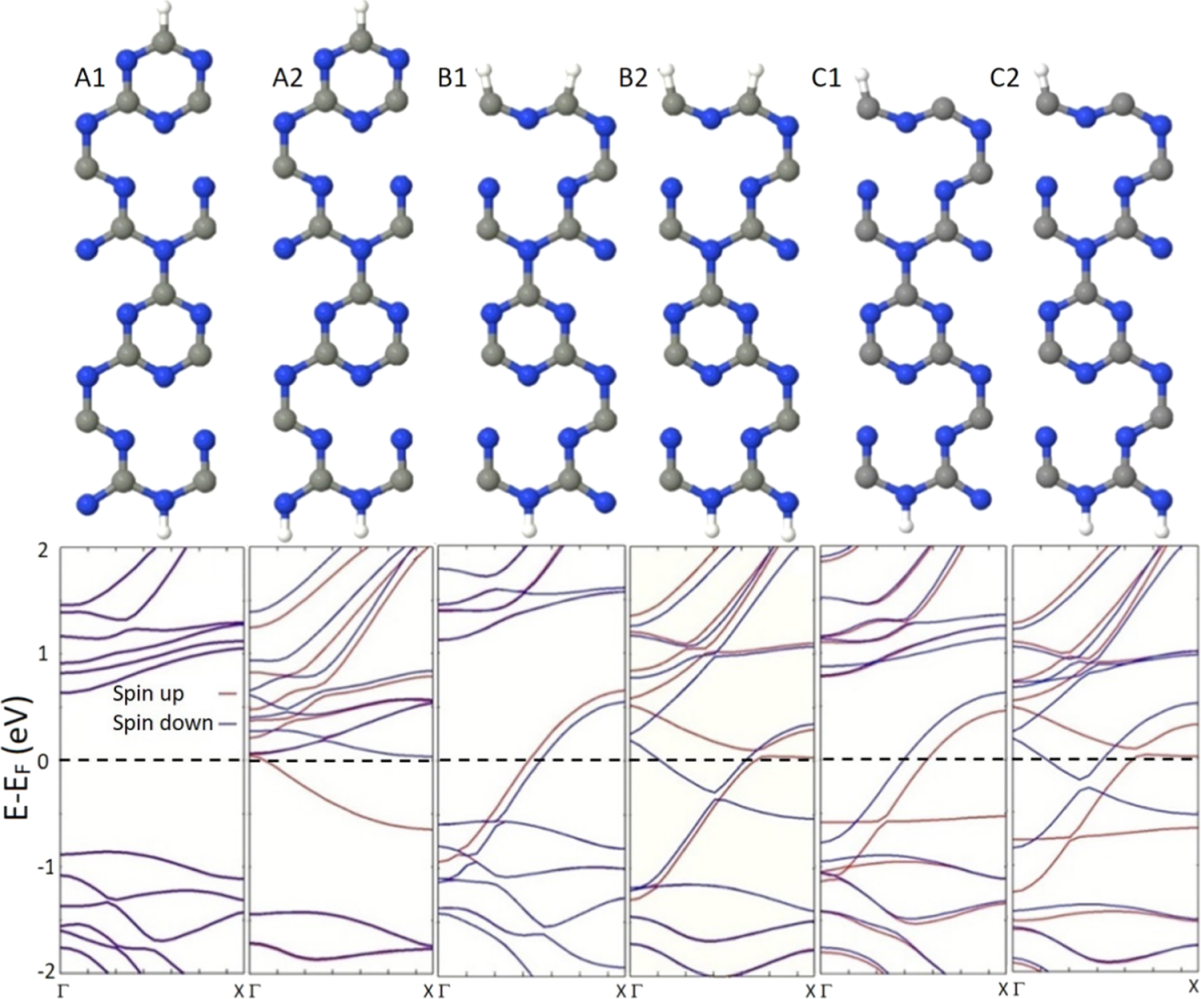
Upper panel illustrates the geometric structures of the exemplary studied gt-CNRs. The lower panel represents the corresponding band structures. The Fermi level is shifted to zero and represented by the black dashed line. C, gray; N, blue; and H, white.

To investigate the structural, electronic, and magnetic properties of gt-CNRs, we employ nanoribbons with different possible carbon-terminated edge structures and H-passivation rates. The optimized exemplary unit cells of the studied configurations are illustrated in the upper panel of Figure 2. It can be deduced from Figure 2 that, unlike structures B1, B2, C1, and C2, the opposite edges of the A1 and A2 systems possess a different topology. First, we examine the energetic stability of the introduced structures by calculating the cohesive energy (***E***_**coh**_) per atom for each configuration using the following equation
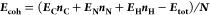
3Here, ***E***_**tot**_ is the total energy of gt-CNR’s unit cell; ***E***_**C**_, ***E***_**N**_, and ***E***_**H**_ are energy per carbon, nitrogen, and hydrogen atoms, respectively; and ***N*** is the number of atoms in the unit cell of gt-CNR. Table 1 summarizes the calculated ***E***_**coh**_ values. It is inferred from Table 1 that structures A1 and A2 exhibit slightly lower energetic stability than structures B1, B2, C1, and C2. It is also deduced from Table 1 that although nanoribbons with low H-passivation rate exhibit better energetic stability, the difference between cohesive energies of similar configurations is negligible.

**Table 1:** Calculated ***E***_**coh**_ Values of the Studied gt-CNRs

structure	A1	A2	B1	B2	C1	C2
***E***_**coh**_ (eV)	0.46	0.45	0.47	0.47	0.49	0.49

**Figure 3:**
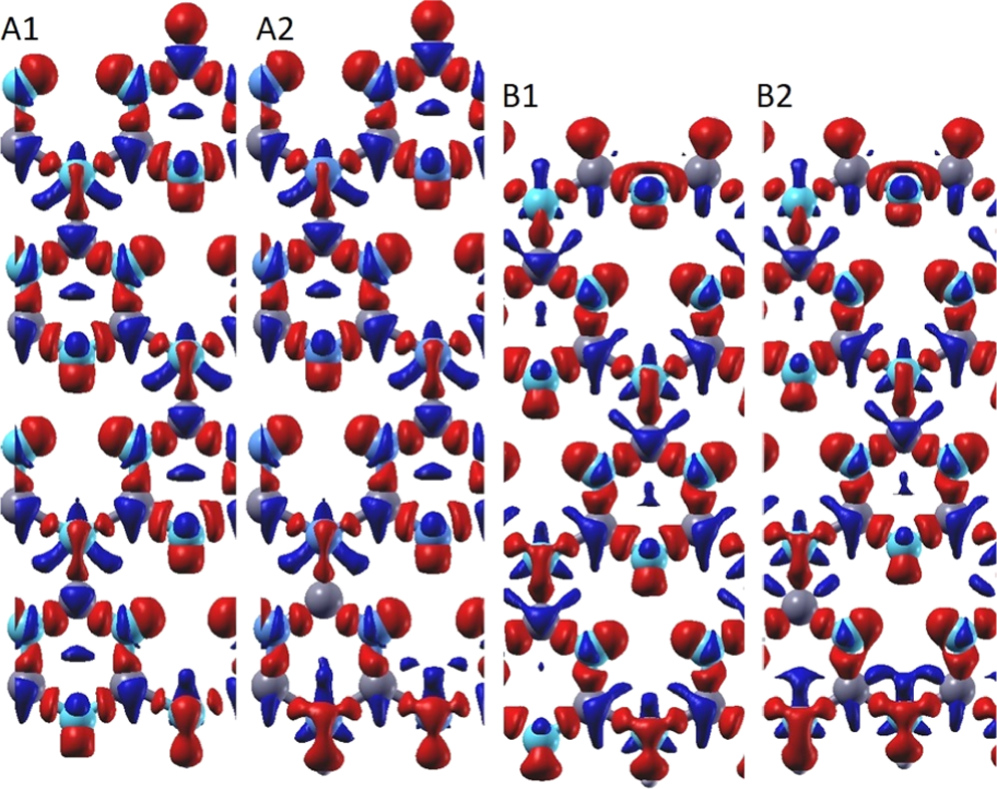
Spin density distribution maps of A1, A2, B1, and B2 structures. C, gray; N, cyan; spin up, red; and spin down, blue.

Since gt-CNRs may host spin-polarized electronic states, it is worthy to investigate the electronic and magnetic structures of the introduced gt-CNRs. To this end, we calculate the spin-polarized band diagrams of the introduced nanoribbons. The lower panel of Figure 2 represents the corresponding band structures of the studied gt-CNRs. It can be seen from the plotted band structures that at a low H-passivation rate, structure A1 exhibits a nonmagnetic electronic structure with an indirect band gap of 1.55 eV, while other systems possessing a metallic band diagram with spin-polarized states in the vicinity of the Fermi level. At a high H-passivation rate, although all systems show spin-polarized band structures, only structure A2 exhibits a half-metallic feature while other nanoribbons show metallic characteristics. This feature is maintained regardless of the width of nanoribbons as discussed in the Supporting Information (SI) and illustrated in Figure S1. For further investigation of the electronic and magnetic properties of gt-CNR, we calculate and plot spin densities throughout the suggested structures in their final magnetic ordering as depicted in Figure 3. Since structures C1 and C2 exhibit band diagrams similar to those of structures B1 and B2, we only represent spin density distributions for structures A1, A2, B1, and B2. As can be deduced from Figure 3, the spin density appears to be distributed almost periodically along the nanoribbons’ width. This is different from the spin density distribution patterns observed in pristine or defected GNRs.^39,84^ Our calculations also show that regardless of different initial spin orientations, i.e., FM or antiferromagnetic (AFM) ordering between opposite edges of studied gt-CNRs, the converged magnetic ordering after self-consistent iterations is always FM. This is an interesting finding since it would be advantageous from the practical viewpoint to design and provide a gt-CNR-based spintronic system with a solid magnetic configuration. It is also worth mentioning that based on our calculations, the favored FM configuration of gt-CNRs is preserved for various widths of the nanoribbon.

**Figure 4:**
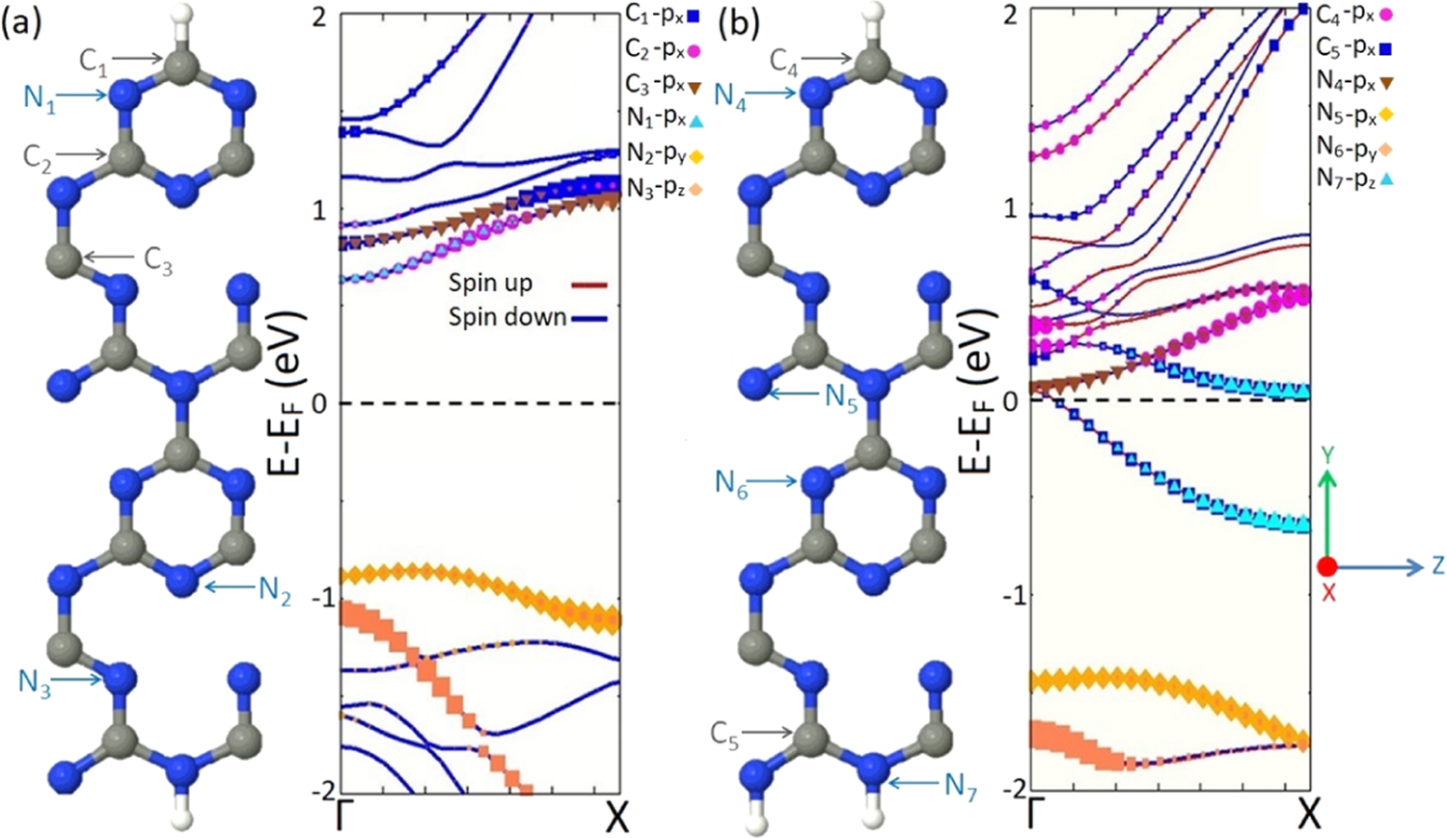
Orbital resolved band structures of systems (a) A1 and (b) A2. The Fermi level is shifted to zero energy and indicated by the black dashed line. C, gray; N, blue; and H, white.

To provide a more detailed insight into the origin of the observed half metallic feature of gt-CNRs, we calculate and compare the orbital resolved electronic structures of systems A1 and A2. As illustrated in Figure 4a, the conduction band (CB) of structure A1 is mainly formed by p*x* orbitals of C and N atoms close to the carbon-terminated edge (atoms C_1_, C_2_, C_3_, and N_1_), while p*y* and p*z* orbitals of N atoms in the vicinity of the nitrogen-terminated edge (atoms N_2_ and N_3_) construct the valence band (VB). For structure A2, it is p*x* and p*z* orbitals of C and N atoms on both edges (atoms C_4_, C_5_, N_4_, and N_7_ as depicted in Figure 4b) that engage in the construction of the CB. The VB of structure A2 is also formed by p*x* and p*y* orbitals of N atoms in the middle of nanoribbon (atoms N_5_ and N_6_). A comparison of the orbital resolved band structures of two systems reveals that the hydrogenation of unsaturated edge N atoms in structure A2 changes the position of atoms and the type of orbitals involved in the formation of the CB and VB. The advent of the half-metallic characteristic in the electronic structure of system A2 can be explained as follows: unsaturated edge nitrogen atoms in system A1 that possess localized lone pair electrons form σ bonding by the hydrogenation in system A2 and therefore inject additional electrons into its electronic structure. These additional electrons are transferred to the edge C and N atoms, occupying the energy states near the Fermi level of system A2 and resulting in the emergence of half metallicity.

**Figure 5:**
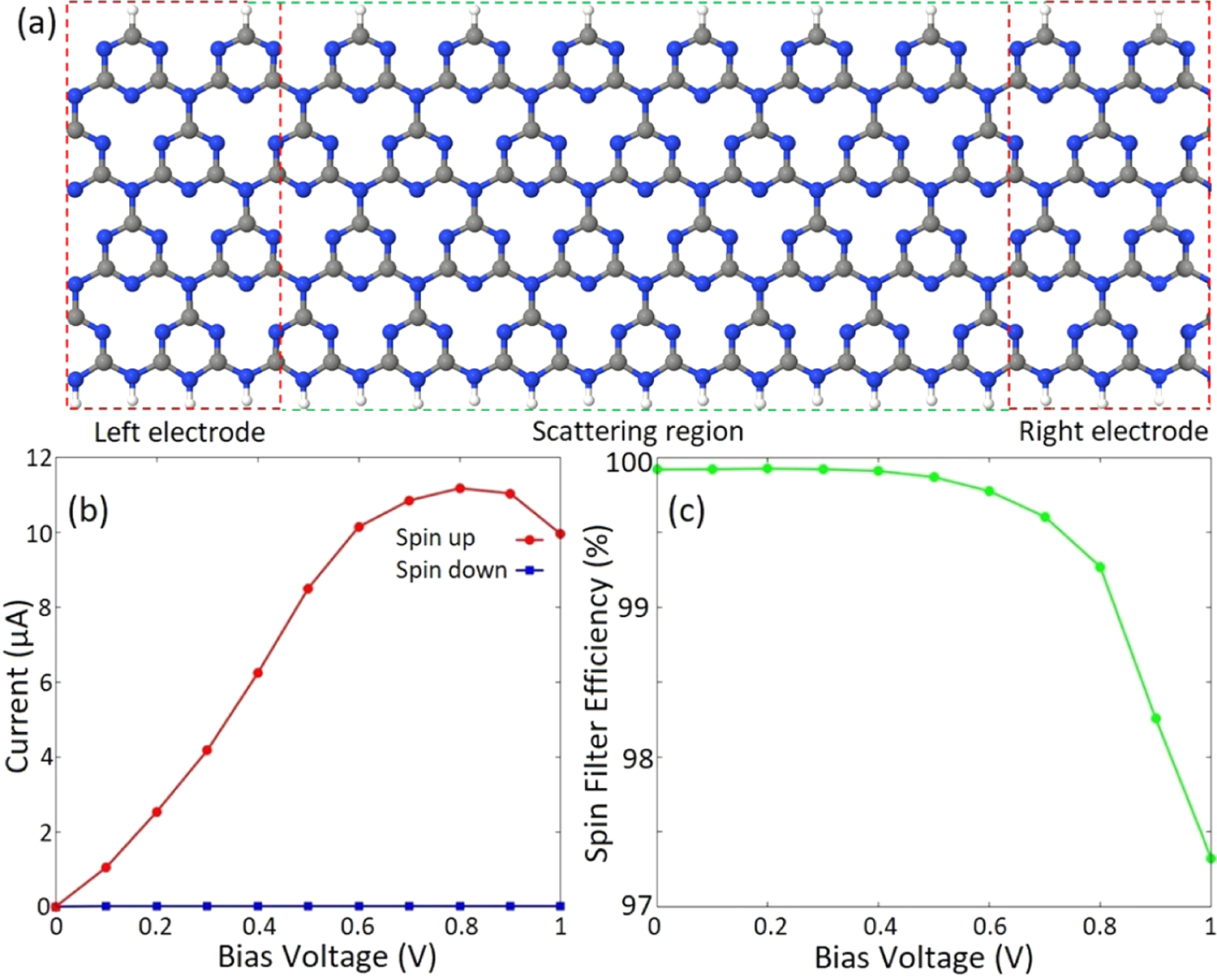
(a) Schematic illustrations of the proposed two-probe transport system. The electrodes and the scattering region are indicated by red and green dashed lines, respectively. C, gray; N, blue; and H, white. (b) Spin-resolved current–voltage and (c) spin filter efficiency profiles of the transport systems.

Given the intrinsic half metallicity of gt-CNR, we investigate its spin-transport properties under a finite bias voltage (*V*_b_) to develop a feasible spin filter device. Figure 5a represents a two-probe system employed to investigate spin-resolved transport features of half-metal gt-CNRs. The calculated current–voltage (*I*–*V*) profiles are illustrated in Figure 5b. It is worth noting that the *V*_b_ range can be chosen with respect to the band gap discrepancies of spin components to obtain the desired proportion of spin filtering. Here, we choose the *V*_b_ range of 0–1 V to achieve the desired spin filtering effect. The selected bias window is also a proper choice from the application viewpoint since it is not large enough to modify the geometrical structure of the device. It is followed from Figure 5b that in the range of applied *V*_b_, the spin-up component shows a nonzero current, while the transport channel for carriers of down spin is blocked. To provide more insight into the charge transport characteristics of the proposed spin filter system, the transmission profiles of the introduced two-probe system are presented in SI, Figure S2 for *V*_b_ = 0 and 0.5 V. Figure 5b also shows that while the spin-down current remains negligible with increasing *V*_b_, the spin-up current increases and reaches its maximum value of 11.1 μA at *V*_b_ = 0.8 V. However, further increase in *V*_b_ suppresses the spin-up current and drops to almost 10 μA at *V*_b_ = 1.0 V. This feature may be due to the decrease in the slope of the spin-up band dispersion as it can be seen in Figure 4b. This indicates that in addition to the spin filter feature, the introduced structure also shows spin negative differential resistance (NDR).^85^ To evaluate the spin filtering capability of the proposed spin filter device, we calculate the SFE of the proposed system. The SFE at a given bias voltage is defined as
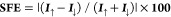
4where ***I***_↑_ and ***I***_↓_ are the spin-up and spin-down currents, respectively. At *V*_b_ = 0 V, the SFE can be obtained by replacing the current values with the corresponding transmission coefficients. Figure 5c represents the calculated SFE.

It shows that the device provides a fully spin-filtered current for *V*_b_ values up to 0.4 V. By increasing *V*_b_, SFE decreases and eventually drops to almost 97.4% at *V*_b_ = 1 V, which is still a significant efficiency. It is worth noting that the SFE of the device is different at higher *V*_b_ values where the bias window may exceed the band gaps of spin-up and spin-down states and consequently the current might be nonzero for both spin components. This feature provides an opportunity to use the proposed system as a nanosized on/off spintronic switch in which the spin filter characteristic of the device can be adjusted by the applied *V*_b_.

## Conclusions

In summary, we have performed a systematic study on the structure, electronic, and magnetic characteristics of triazine-based graphitic carbon nitride nanoribbons using the first-principles calculations. To this end, we first examine the energetic stability of gt-CNRs with possible edge structures and H-passivation rates. Our calculations show that edge configuration and hydrogenation rate affect, albeit not significantly, the stability of gt-CNRs. Next, by calculating spin-polarized band diagrams, we show that electronic and magnetic characteristics of the studied nanoribbons depend on their edge structures and H-passivation rate in which only gt-CNRs with a certain edge configuration and fully hydrogenated edges exhibit the half-metallic feature. Plotting calculated spin density distribution over various gt-CNRs reveals that FM ordering is a robust feature of gt-CNRs. To investigate the origin of the observed half metallicity, we calculate and compare the orbital resolved band structures of NM and half-metal gt-CNRs. It is shown that the hydrogenation of unsaturated edge N atoms injects additional electrons into the electronic structure of gt-CNR and hence provides an energy level under the CB of gt-CNR in the spin-up channel. To investigate the possible spintronic applications of gt-CNRs, we study the spin-transport properties of a two-probe system composed of a half-metal gt-CNR. The performed calculations show that the proposed spin-transport system possesses an SFE of 100% for a practically feasible *V*_b_ range. It is also shown that aside from its spin filtering feature, the introduced two-probe system exhibits NDR as well. Having all the mentioned practical and robust characteristics, the gt-CNR-based spin filter system is a promising candidate to achieve a feasible multifunctional atomically thin spintronic device.

## Supporting Information Available

Electronic and magnetic structures of gt-CNRs; width effect; spin-polarized band diagrams and transmission profiles; half-metallic gt-CNR; and spin filtering effect (PDF)

## References

[ref1] Wolf S. A.; Awschalom D. D.; Buhrman R. A.; Daughton J. M.; von Molnár S.; Roukes M. L.; Chtchelkanova A. Y.; Treger D. M. Spintronics: A Spin-Based Electronics Vision for the Future. Science 2001, 294, 1488–1495. 10.1126/science.1065389. 11711666

[ref2] Han W.; Kawakami R. K.; Gmitra; Fabian M. J. Graphene spintronics. Nat. Nanotechnol. 2014, 9, 794–807. 10.1038/nnano.2014.214. 25286274

[ref3] Awschalom D. D.; Flatt M. E. Challenges for semiconductor spintronics,. Nat. Phys. 2007, 3, 153–159. 10.1038/nphys551.

[ref4] Felser C.; Fecher G. H.; Balke B. Spintronics: a challenge for materials science and solid-state chemistry. Angew. Chem., Int. Ed. 2007, 46, 668–699. 10.1002/anie.200601815. 17219604

[ref5] Zhou J.; Sun Q. Magnetism of Phthalocyanine-Based Organometallic Single Porous Sheet. J. Am. Chem. Soc. 2011, 133, 15113–15119. 10.1021/ja204990j. 21838296

[ref6] Kan E.; Hu W.; Xiao C.; Lu R.; Deng K.; Yang J.; Su H. Half-Metallicity in Organic Single Porous Sheets. J. Am. Chem. Soc. 2012, 134, 5718–5721. 10.1021/ja210822c. 22440006

[ref7] Wu F.; Huang C.; Wu H.; Lee C.; Deng K.; Kan E.; Jena P. Atomically Thin Transition-Metal Dinitrides: High-Temperature Ferromagnetism and Half-Metallicity. Nano Lett. 2015, 15, 8277–8281. 10.1021/acs.nanolett.5b03835. 26575002

[ref8] Ashton M.; Gluhovic D.; Sinnott S. B.; Guo J.; Stewart D. A.; Henning R. G. Two-Dimensional Intrinsic Half-Metals with Large Spin Gaps,. Nano Lett. 2017, 17, 5251–5257. 10.1021/acs.nanolett.7b01367. 28745061

[ref9] Li X.; Lv H.; Dai J.; Ma L.; Zeng X. C.; Wu X.; Yang J. Half Metallicity in One-Dimensional Metal Trihydride Molecular Nanowires. J. Am. Chem. Soc. 2017, 139, 6290–6293. 10.1021/jacs.7b01369. 28453273

[ref10] Yao X.; Yuan S.; Wang J. Theoretical Studies of Sandwich Molecular Wires with Europium and Boratacyclooctatetraene Ligand and the Structure on a H-Ge(001)-2 × 1 Surface. J. Phys. Chem. C 2016, 120, 7088–7093. 10.1021/acs.jpcc.5b11660.

[ref11] Zhang X.; Wang J.; Gao Y.; Zeng X. C. Ab Initio Study Of Structural and Magnetic Properties of TM_n_(Ferrocene)_n+1_ (TM = Sc, Ti, V, Mn) Sandwich Clusters and Nanowires (n = ∞). ACS Nano 2009, 3, 537–545. 10.1021/nn800794c. 19256546

[ref12] Wan Y.; Sun Y.; Wu X.; Yang J. Ambipolar Half-Metallicity in One-Dimensional Metal-(1,2,4,5- Benzenetetramine) Coordination Polymers via Carrier Doping. J. Phys. Chem. C 2018, 122, 989–994. 10.1021/acs.jpcc.7b12022.

[ref13] Novoselov K. S.; Fal′ko V. I.; Colombo L.; Gellert P. R.; Schwab M. G.; Kim K. A Roadmap for Graphene. Nature 2012, 490, 192–200. 10.1038/nature11458. 23060189

[ref14] Bhimanapati G. R.; Lin Z.; Meunier V.; et al. Recent Advances in Two-Dimensional Materials beyond Graphene. ACS Nano 2015, 9, 11509–11539. 10.1021/acsnano.5b05556. 26544756

[ref15] Rezapour M. R.; Myung C. W.; Yun J.; Ghassami A.; Li N.; Yu S. U.; Hajibabaei A.; Park Y.; Kim K. S. Graphene and Graphene Analogs toward Optical, Electronic, Spintronic, Green-Chemical, Energy-Material, Sensing, and Medical Applications. ACS Appl. Mater. Interfaces 2017, 9, 24393–24406. 10.1021/acsami.7b02864. 28678466

[ref16] Feliciano G. T.; Sanz-Navarro C.; Coutinho-Neto M. D.; Ordejon P.; Scheicher R. H.; Rocha A. R. Capacitive DNA Detection Driven by Electronic Charge Fluctuations in a Graphene Nanopore. Phys. Rev. Appl. 2015, 3, 034003 10.1103/PhysRevApplied.3.034003.

[ref17] Kim S. W.; Kim H. J.; Choi J. H.; Scheicher R. H.; Cho J. H. Contrasting Interedge Superexchange Interactions of Graphene Nanoribbons Embedded in h-BN and Graphane. Phys. Rev. B 2015, 92, 035443 10.1103/PhysRevB.92.035443.

[ref18] Cresti A.; Nemec N.; Biel B.; Niebler G.; Triozon F.; Cuniberti G.; Roche S. Charge Transport in Disordered Graphene-Based Low Dimensional Materials. Nano Res. 2008, 1, 361–394. 10.1007/s12274-008-8043-2.

[ref19] González C.; Dappe Y. J.; Biel B. Reactivity Enhancement and Fingerprints of Point Defects on a MoS_2_ Monolayer Assessed by ab Initio Atomic Force Microscopy. J. Phys. Chem. C 2016, 120, 17115–17126. 10.1021/acs.jpcc.6b05998.

[ref20] Chen Y.; Tan C.; Zhang H.; Wang L. Two-Dimensional Graphene Analogues for Biomedical Applications. Chem. Soc. Rev. 2015, 44, 2681–2701. 10.1039/C4CS00300D. 25519856

[ref21] Avdoshenko S. M.; Nozaki D.; Gomes da Rocha C.; González J. W.; Lee M. H.; Gutierrez R.; Cuniberti G. Dynamic and Electronic Transport Properties of DNA Translocation through Graphene Nanopores. Nano Lett. 2013, 13, 1969–1976. 10.1021/nl304735k. 23586585

[ref22] Rao C. N. R.; Matte H. S. S. R.; Maitra U. Graphene Analogues of Inorganic Layered Materials. Angew. Chem., Int. Ed. 2013, 52, 13162–13185. 10.1002/anie.201301548. 24127325

[ref23] Wu T. T.; Wang X. F.; Zhai M. X.; Liu H.; Zhou L.; Jiang Y. J. Negative Differential Spin Conductance in Doped Zigzag Graphene Nanoribbons. Appl. Phys. Lett. 2012, 100, 052112 10.1063/1.3681775.

[ref24] Yang X. F.; Zhou W. Q.; Hong X. K.; Liu Y. S.; Wang X. F.; Feng J. F. Half-Metallic Properties, Single-Spin Negative Differential Resistance, and Large Single-Spin Seebeck effects Induced by chemical Doping in Zigzag-Edged Graphene Nanoribbons. J. Chem. Phys. 2015, 142, 024706 10.1063/1.4904295. 25591376

[ref25] Zheng X. H.; Rungger I.; Zeng Z.; Sanvito S. Effects Induced by Single and Multiple Dopants on the Transport Properties in Zigzagedged Graphene Nanoribbons. Phys. Rev. B 2009, 80, 235426 10.1103/PhysRevB.80.235426.

[ref26] Munárriz J.; Gaul C.; Malyshev A. V.; Orellana P. A.; Müller C. A.; Domínguez-Adame F. Strong Spin-Dependent Negative Differential Resistance in Composite Graphene Superlattices. Phys. Rev. B 2013, 88, 155423 10.1103/PhysRevB.88.155423.

[ref27] Pisani L.; Chan J. A.; Montanari B.; Harrison N. M. Electronic Structure and Magnetic Properties of Graphitic Ribbons. Phys. Rev. B 2007, 75, 064418 10.1103/PhysRevB.75.064418.

[ref28] Barone V.; Hod O.; Scuseria G. E. Electronic Structure and Stability of Semiconducting Graphene Nanoribbons. Nano Lett. 2006, 6, 2748–2754. 10.1021/nl0617033. 17163699

[ref29] Biel B.; Blasé X.; Triozon F.; Roche S. Anomalous Doping Effects on Charge Transport in Graphene Nanoribbons. Phys. Rev. Lett. 2009, 102, 096803 10.1103/PhysRevLett.102.096803. 19392549

[ref30] Son Y. W.; Cohen M. L.; Louie S. G. Energy Gaps in Graphene Nanoribbons. Phys. Rev. Lett. 2006, 97, 216803 10.1103/PhysRevLett.97.216803. 17155765

[ref31] Son Y. W.; Cohen M. L.; Louie S. G. Half-metallic graphene nanoribbons. Nature 2006, 444, 347–349. 10.1038/nature05180. 17108960

[ref32] Shin Y. S.; Son J. Y.; Jo M. H.; Shin Y. H.; Jang H. M. High Mobility Graphene Nanoribbons Prepared Using Polystyrene Dip-Pen Nanolithography. J. Am. Chem. Soc. 2011, 133, 5623–5625. 10.1021/ja108464s. 21443183

[ref33] Rajan A. C.; Rezapour M. R.; Yun J.; Cho Y.; Cho W. J.; Min S. K.; Lee G.; Kim K. S. Two Dimensional Molecular Electronics Spectroscopy for Molecular Fingerprinting, DNA Sequencing, and Cancerous DNA Recognition. ACS Nano 2014, 8, 1827–1833. 10.1021/nn4062148. 24446806

[ref34] Marconcini P.; Cresti A.; Triozon F.; Fiori G.; Biel B.; Niquet Y. M.; Macucci M.; Roche S. Atomistic Boron-Doped Graphene Field-Effect Transistors: A Route toward Unipolar Characteristics. ACS Nano 2012, 6, 7942–7947. 10.1021/nn3024046. 22876866

[ref35] Rezapour M. R.; Lee G.; Kim K. S. An Effective Approach to Realize Graphene Based p-n Junctions via Adsorption of Donor and Acceptor Molecules. Carbon 2019, 153, 525–530. 10.1016/j.carbon.2019.07.062.

[ref36] Prasongkit J.; Grigoriev A.; Pathak B.; Ahuja R.; Scheicher R. H. Transverse Conductance of DNA Nucleotides in a Graphene Nanogap from First Principles. Nano Lett. 2011, 11, 1941–1945. 10.1021/nl200147x. 21495701

[ref37] Rezapour M. R.; Rajan A. C.; Kim K. S. Molecular Sensing using Armchair Graphene Nanoribbon. J. Comput. Chem. 2014, 35, 1916–1920. 10.1002/jcc.23705. 25117934

[ref38] Muñoz-Rojas F.; Fernandez-Rossier J.; Palacios J. J. Giant Magnetoresistance in Ultrasmall Graphene Based Devices. Phys. Rev. Lett. 2009, 102, 136810 10.1103/PhysRevLett.102.136810. 19392393

[ref39] Rezapour M. R.; Lee G.; Kim K. S. A High Performance N-doped Graphene Nanoribbon Based Spintronic Device Applicable with a Wide Range of Adatoms. Nanoscale Adv. 2020, 2, 5905–5911. 10.1039/D0NA00652A. 36133856 PMC9419213

[ref40] Rezapour M. R.; Yun J.; Lee G.; Kim K. S. Lower Electric Field-Driven Magnetic Phase Transition and Perfect Spin Filtering in Graphene Nanoribbons by Edge Functionalization. J. Phys. Chem. Lett. 2016, 7, 5049–5055. 10.1021/acs.jpclett.6b02437. 27973868

[ref41] Kan E. J.; Li Z.; Yang J.; Hou J. G. Half-Metallicity in Edge-Modified Zigzag Graphene Nanoribbons. J. Am. Chem. Soc. 2008, 130, 4224–4225. 10.1021/ja710407t. 18331034

[ref42] Dutta S.; Manna A. K.; Pati S. K. Intrinsic Half-Metallicity in Modified Graphene Nanoribbons. Phys. Rev. Lett. 2009, 102, 096601 10.1103/PhysRevLett.102.096601. 19392544

[ref43] Pereira V. M.; Guinea F.; Lopes dos Santos J. M. B.; Peres N. M. R.; Castro Neto A. H. Disorder Induced Localized States in Graphene. Phys. Rev. Lett. 2007, 96, 036801 10.1103/PhysRevLett.96.036801. 16486750

[ref44] Li C.; Xu Y.; Tu W.; Chen G.; Xu R. Metal-free Photocatalysts for Various Applications in Energy Conversion and Environmental Purification. Green Chem. 2017, 19, 882–899. 10.1039/C6GC02856J.

[ref45] Liu G.; Zhao G.; Zhou W.; Liu Y.; Pang H.; Zhang H.; Hao D.; Meng X.; Li P.; Kako T.; Ye J. In Situ Bond Modulation of Graphitic Carbon Nitride to Construct p-n Homojunctions for Enhanced Photocatalytic Hydrogen Production. Adv. Funct. Mater. 2016, 26, 6822–6829. 10.1002/adfm.201602779.

[ref46] Wang J. C.; Cui C. X.; Kong Q. Q.; Ren C. Y.; Li Z.; Qu L.; Zhang Y.; Jiang K. Doped g-C_3_N_4_ Nanoribbon for Efficient Visible-Light Photocatalytic Water Splitting Coupling with Methylene Blue Degradation. ACS Sustainable Chem. Eng. 2018, 6, 8754–8761. 10.1021/acssuschemeng.8b01093.

[ref47] Zhang X.; Zhao M.; Wang A.; Wang X.; Du A. Spin-Polarization and Ferromagnetism of Graphitic Carbon Nitride Materials. J. Mater. Chem. C 2013, 1, 6265–6270. 10.1039/c3tc31213e.

[ref48] Yu H.; Jiang X.; Shao Z.; Feng J.; Yang X.; Liu Y. Metal-Free Half-Metallicity in B-Doped gh-C_3_N_4_ Systems. Nanoscale Res. Lett. 2018, 13, 57 10.1186/s11671-018-2473-x. 29464414 PMC5820242

[ref49] Meng B.; Xiao W. Z.; Wang L. L.; Yue L.; Zhang S.; Zhang H. Y. Half-Metallic and Magnetic Properties in Nonmagnetic Element Embedded Graphitic Carbon Nitride Sheets. Phys. Chem. Chem. Phys. 2015, 17, 22136–22143. 10.1039/C5CP03794H. 26256953

[ref50] Choudhuri I.; Kumar S.; Mahata A.; Rawat K. S.; Pathak B. Transition-Metal Embedded Carbon Nitride Monolayers: High-Temperature Ferromagnetism and Half-Metallicity. Nanoscale 2016, 8, 14117–14126. 10.1039/C6NR03282F. 27321785

[ref51] Yang S.; Gong Y.; Zhang J.; Zhan L.; Ma L.; Fang Z.; Vajtai R.; Wang X.; Ajayan P. M. Exfoliated Graphitic Carbon Nitride Nanosheets as Efficient Catalysts for Hydrogen Evolution under Visible Light. Adv. Mater. 2013, 25, 2452–2456. 10.1002/adma.201204453. 23450777

[ref52] Zhang X.; Xie X.; Wang H.; Zhang J.; Pan B.; Xie Y. Enhanced Photoresponsive Ultrathin Graphitic-Phase C_3_N_4_ Nanosheets for Bioimaging. J. Am. Chem. Soc. 2013, 135, 18–21. 10.1021/ja308249k. 23244197

[ref53] Du A. J.; Sanvito S.; Li Z.; Wang D. W.; Jiao Y.; Liao T.; Sun Q.; Ng Y. H.; Zhu Z. H.; Amal R.; Smith S. C. Hybrid Graphene and Graphitic Carbon Nitride Nanocomposite: Gap Opening, Electron–Hole Puddle, Interfacial Charge Transfer, and Enhanced Visible Light Response. J. Am. Chem. Soc. 2012, 134, 4393–4397. 10.1021/ja211637p. 22339061

[ref54] Wang X. C.; Maeda K.; Thomas A.; Takanabe K.; Xin G.; Carlsson J. M.; Domen K.; Antonietti M. A metal-Free Polymeric Photocatalyst for Hydrogen Production from Water under Visible Light. Nat. Mater. 2009, 8, 76–80. 10.1038/nmat2317. 18997776

[ref55] Groenewolt M.; Antonietti M. Synthesis of g-C_3_N_4_ Nanoparticles in Mesoporous Silica Host Matrices. Adv. Mater. 2005, 17, 1789–1792. 10.1002/adma.200401756.

[ref56] Wang X.; Chen X.; Thomas A.; Fu X.; Antonietti M. Metal-Containing Carbon Nitride Compounds: A New Functional Organic–Metal Hybrid Material. Adv. Mater. 2009, 21, 1609–1612. 10.1002/adma.200802627.

[ref57] Thomas A.; Fischer A.; Goettmann F.; Antonietti M.; Müller J. O.; Schlögl R.; Carlsson J. M. Graphitic Carbon Nitride Materials: Variation of Structure and Morphology and Their use as Metal-Free Catalysts. J. Mater. Chem. 2008, 18, 4893–4908. 10.1039/b800274f.

[ref58] Zhu J.; Xiao P.; Li H.; Carabineiro S. A. Graphitic Carbon Nitride: Synthesis, Properties, and Applications in Catalysis. ACS Appl. Mater. Interfaces 2014, 6, 16449–16465. 10.1021/am502925j. 25215903

[ref59] Zheng Y.; Lin L.; Wang B.; Wang X. Graphitic Carbon Nitride Polymers toward Sustainable Photoredox Catalysis. Angew. Chem., Int. Ed. 2015, 54, 12868–12884. 10.1002/anie.201501788. 26424620

[ref60] Wen J.; Xie J.; Chen X.; Li X. A Review on g-C_3_N_4_-Based Photocatalysts. Appl. Surf. Sci. 2017, 391, 72–123. 10.1016/j.apsusc.2016.07.030.

[ref61] Ghosh D.; Periyasamy G.; Pandeyc B.; Pati S. K. Computational Studies on Magnetism and the Optical Properties of Transition Metal Embedded Graphitic Carbon Nitride Sheets. J. Mater. Chem. C 2014, 2, 7943–7951. 10.1039/C4TC01385A.

[ref62] Choudhuri I.; Bhattacharyya G.; Kumar S.; Pathak B. Metal-Free Half-Metallicity in a High Energy Phase C-Doped gh-C_3_N_4_ System: A High Curie Temperature Planar System. J. Mater. Chem. C 2016, 4, 11530–11539. 10.1039/C6TC04163A.

[ref63] Fang L.; Ohfuji H.; Shinmei T.; Irifune T. Experimental Study on the Stability of Graphitic C_3_N_4_ under High Pressure and High Temperature. Diamond Relat. Mater. 2011, 20, 819–825. 10.1016/j.diamond.2011.03.034.

[ref64] Kroke E.; Schwarz M.; Horath-Bordon E.; Kroll P.; Noll B.; Norman A. D. Tri-s-triazine derivatives. Part I. From trichloro-tri-s-triazine to graphitic C_3_N_4_ structures. New J. Chem. 2002, 26, 508–512. 10.1039/b111062b.

[ref65] Kroke E. gt-C3N4-The First Stable Binary Carbon (IV) Nitride. Angew. Chem., Int. Ed. 2014, 53, 11134–11136. 10.1002/anie.201406427. 25168265

[ref66] Liu Y.; Liu P.; Sun C.; Wang T.; Tao K.; Gao D. P Dopants Induced Ferromagnetism in g-C_3_N_4_ Nanosheets: Experiments and Calculations. Appl. Phys. Lett. 2017, 110, 222403 10.1063/1.4984584.

[ref67] Du A. J.; Sanvito S.; Smith S. C. First-Principles Prediction of Metal-Free Magnetism and Intrinsic Half-Metallicity in Graphitic Carbon Nitride. Phys. Rev. Lett. 2012, 108, 197–207. 10.1103/PhysRevLett.108.197207. 23003085

[ref68] Gao D.; Xu Q.; Zhang J.; Yang Z.; Si M.; Yan Z.; Xue D. Defect-Related Ferromagnetism in Ultrathin Metal-Free g-C_3_N_4_ Nanosheets. Nanoscale 2014, 6, 2577–2581. 10.1039/c3nr04743a. 24464248

[ref69] Xu K.; Li X. L.; Chen P. Z.; Zhou D.; Wu C. Z.; Guo Y. Q.; Zhang L. D.; Zhao J. Y.; Wu X. J.; Xie Y. Hydrogen Dangling Bonds Induce Ferromagnetism in Two-Dimensional Metal-Free Graphitic-C_3_N_4_ Nanosheets. Chem. Sci. 2015, 6, 283–287. 10.1039/C4SC02576H. 28580096 PMC5435869

[ref70] Gao D.; Liu Y.; Song M.; Shi S.; Si M.; Xue D. Manifestation of High-Temperature Ferromagnetism in Fluorinated Graphitic Carbon Nitride Nanosheets. J. Mater. Chem. C 2015, 3, 12230–12235. 10.1039/C5TC02911B.

[ref71] Gao D.; Liu Y.; Liu P.; Si M.; Xue D. Atomically Thin B Doped gC_3_N_4_ Nanosheets: High-Temperature Ferromagnetism and Calculated Half-Metallicity. Sci. Rep. 2016, 6, 35768 10.1038/srep35768. 27762348 PMC5071904

[ref72] Zhao Y.; Zhao F.; Wang X.; Xu C.; Zhang Z.; Shi G.; Qu L. Graphitic Carbon Nitride Nanoribbons: Graphene-Assisted Formation and Synergic Function for Highly Efficient Hydrogen Evolution. Angew. Chem., Int. Ed. 2014, 53, 13934–13939. 10.1002/anie.201409080. 25381722

[ref73] Bu X.; Bu Y.; Yang S.; et al. Graphitic Carbon Nitride Nanoribbon for Enhanced Visible-Light Photocatalytic H_2_ production. RSC Adv. 2016, 6, 112210–112214. 10.1039/C6RA23218C.

[ref74] Liu Y.; Guo X.; Chen Z.; Zhang W.; Wang Y.; Zheng Y.; Tang X.; Zhang M.; et al. Microwave-Synthesis of g-C_3_N_4_ Nanoribbons Assembled Seaweed-Like Architecture with Enhanced Photocatalytic Property. Appl. Catal., B 2020, 266, 118624 10.1016/j.apcatb.2020.118624.

[ref75] Kresse G.; Furthmüller J. Efficient Iterative Schemes for ab Initio Total-Energy Calculations Using a Plane-Wave Basis Set. Phys. Rev. B 1996, 54, 11169 10.1103/PhysRevB.54.11169. 9984901

[ref76] Perdew J. P.; Burke K.; Ernzerhof M. Generalized Gradient Approximation Made Simple. Phys. Rev. Lett. 1996, 77, 3865–3868. 10.1103/PhysRevLett.77.3865. 10062328

[ref77] Kresse G.; Joubert D. From Ultrasoft Pseudopotentials to the Projector Augmented-Wave Method. Phys. Rev. B 1999, 59, 1758 10.1103/PhysRevB.59.1758.

[ref78] Datta S. Electronic Transport in Mesoscopic Systems; Cambridge University Press: Cambridge, 1997.

[ref79] Brandbyge M.; Mozos J.-L.; Ordejón P.; Taylor J.; Stokbro K. Density-Functional Method for Nonequilibrium Electron Transport. Phys. Rev. B 2002, 65, 165401 10.1103/PhysRevB.65.165401.

[ref80] Xu L.; Huang W. Q.; Wang L. L.; Tian Z. A.; Hu W.; Ma Y.; Wang X.; Pan A.; Huang G. F. Insights into Enhanced Visible-Light Photocatalytic Hydrogen Evolution of g-C_3_N_4_ and Highly Reduced Graphene Oxide Composite: The Role of Oxygen. Chem. Mater. 2015, 27, 1612–1621. 10.1021/cm504265w.

[ref81] Bafekry A.; Shayesteh S. F.; Peeters F. Two-Dimensional Carbon Nitride (2DCN) Nanosheets: Tuning of Novel Electronic and Magnetic Properties by Hydrogenation, Atom Substitution and Defect Engineering. J. Appl. Phys. 2019, 126, 215104 10.1063/1.5120525.

[ref82] Yao Q.; Lu M.; Du Y.; Wu F.; Deng K.; Kan E. Designing Half-Metallic Ferromagnetism by a New Strategy: An Example of Superhalogen Modified Graphitic C_3_N_4_. J. Mater. Chem. C 2018, 6, 1709–1714. 10.1039/C7TC05087A.

[ref83] Zhu B.; Cheng B.; Zhang L.; Yu J. Review on DFT Calculation of s-Triazine-based Carbon Nitride. Carbon Energy 2019, 1, 32–56. 10.1002/cey2.1.

[ref84] Magda G. Z.; Jin X.; Hagymási I.; Vancsó P.; Osváth Z.; Nemes-Incze P.; Hwang C.; Biró L. P.; Tapasztó L. Room-Temperature Magnetic Order on Zigzag Edges of Narrow Graphene Nanoribbons. Nature 2014, 514, 608–611. 10.1038/nature13831. 25355361

[ref85] Kong X.; Cui B.; Zhao W.; Zhao J.; Li D.; Liu D. Spin Negative Differential Resistance and High Spin Filtering Behavior Realized by Devices Based on Graphene Nanoribbons and Graphitic Carbon Nitrides. Org. Electron. 2014, 15, 3674–3680. 10.1016/j.orgel.2014.10.016.

