# Supplementary material for: Structural, Electronic, and Magnetic Characteristics of Graphitic Carbon Nitride Nanoribbons and Their Applications in Spintronics
Source: J Phys Chem C Nanomater Interfaces. 2022 Sep 15;126(38):16429–36. doi: 10.1021/acs.jpcc.2c04691 (PMC9527752; doi:10.1021/acs.jpcc.2c04691)
Supplement: Supplementary file 1 — jp2c04691_si_001.pdf [file jp2c04691_si_001.pdf]

Supporting Information for

“Structural, Electronic and Magnetic Characteristics of Graphitic Carbon Nitride Nanoribbons and Their Applications in Spintronics”

*M. Reza Rezapour**

Department of Atomic, Molecular and Nuclear Physics, Faculty of Science, Campus de Fuente Nueva, University of Granada, 18071 Granada, Spain.

*email: rezapour@ugr.es

Electronic and magnetic structures of gt-CNRs: The width effect

In order to investigate the effect of the width of gt-CNR on its electronic and magnetic characteristics, we calculate and plot spin polarized band diagrams of gt-CNRs of different widths as shown in Figure S1. It can be inferred from Figure S1 that the characteristics of band structure of gt-CNR are width independent and only are a function of its edge structure and H-passivation rate. As one can see, despite their different widths, gt-CNRs with same edge configurations and H-passivation rates possess similar band structures. This is a notable feature since it expands the range of applicable gt-CNRs and facilitates designing and fabricating gt-CNR based spintronic devices.

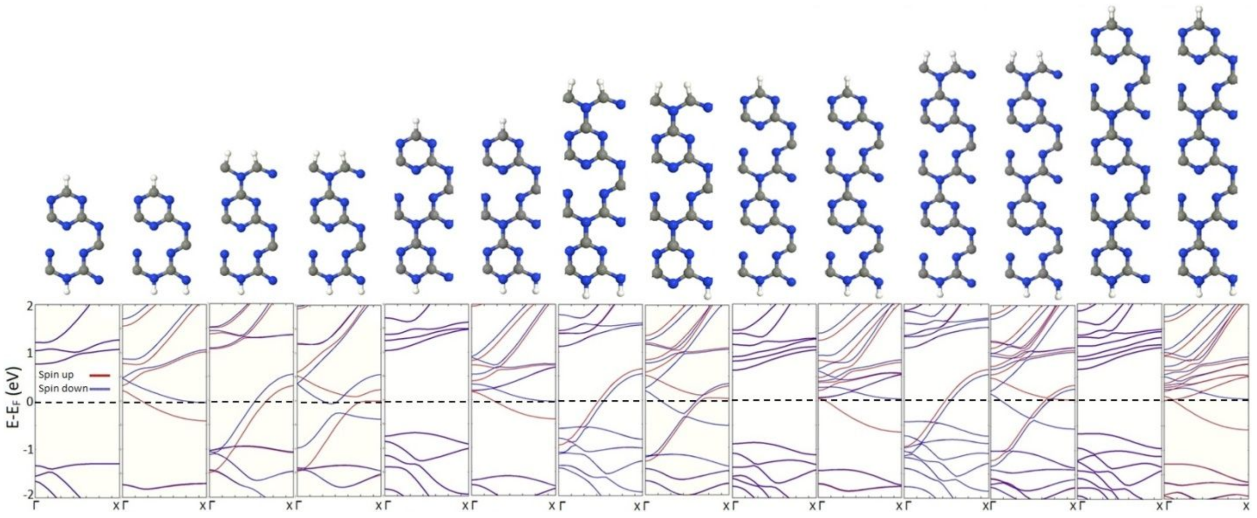

Figure S1: Upper panel: Geometric structures of gt-CNRs with different widths. Lower panel: Corresponding band structures of the depicted gt-CNRs in the upper panel. The Fermi level is shifted to zero and represented by the black dashed line. C; gray, N; blue, and H; white.

Transmission profile of half-metallic gt-CNR

To provide a better understanding on electron transport characteristics of a spin filter composed of half metallic gt-CNR, transmission profiles of the two-probe system illustrated in Figure 5a is presented in Figure S2 for two bias voltage values of V_b = 0 V and 0.5 V. One can see that while spin up component provides non-zero current for various bias voltages, the spin down channel remains almost blocked for practical bias window. However, if at larger V_b values the spin up and down states possess band gaps smaller than the bias window, the current would be non-zero for both spin components which means that the proposed system can be employed as a nano-size on/off spintronic switch where the spin filtering characteristic of the device can be tuned by the applied bias voltage.

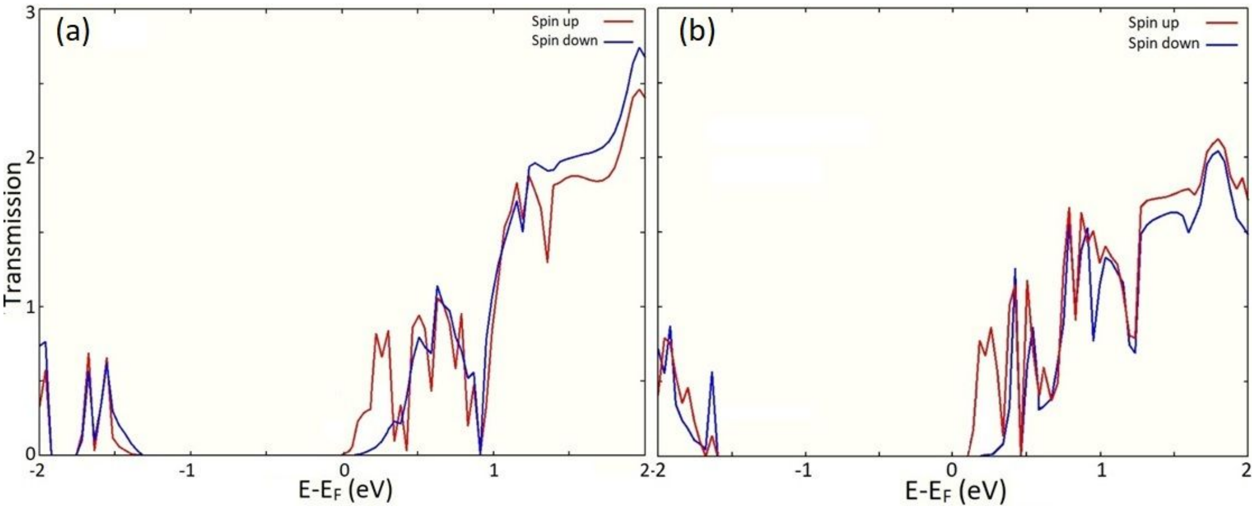

Figure S1: Transmission profile of the proposed two-probe system for (a) V_b = 0 V and (b) 0.5 V
